# Self-Rated Health in the Last 12 Years of Life Compared to Matched Surviving Controls: The Health and Retirement Study

**DOI:** 10.1371/journal.pone.0107879

**Published:** 2014-09-19

**Authors:** Sari Stenholm, Jaana Pentti, Ichiro Kawachi, Hugo Westerlund, Mika Kivimäki, Jussi Vahtera

**Affiliations:** 1 Department of Public Health, University of Turku, Turku, Finland; 2 Finnish Institute of Occupational Health, Helsinki, Finland; 3 Department of Society, Human Development, and Health, Harvard School of Public Health, Boston, Massachusetts, United States of America; 4 Stress Research Institute, Stockholm University, Stockholm, Sweden; 5 Department of Epidemiology and Public Health, University College London, London, United Kingdom; 6 Hjelt Institute, Medical Faculty, University of Helsinki, Helsinki, Finland; 7 Turku University Hospital, Turku, Finland; Oregon Health & Science University, United States of America

## Abstract

Self-rated health (SRH) is a valid measure of health status and associated with mortality. Based on individual-level biannual repeat data on SRH we sought to characterize the natural history of poor SRH during the 12 years prior to death in men and women in different age groups. We conducted a retrospective analysis of the Health and Retirement Study participants who died between 1998 and 2010 and had at least two SRH measurements in the 12 years prior to death. We used a nested case-control design to compare SRH trajectories of deceased men and women aged 30–64, 65–79 and 80 years versus surviving participants. The cases comprised 3,350 deceased participants who were matched to surviving controls (n = 8,127). SRH was dichotomized into good vs. poor health. Men and women dying at age 65–79 and ≥80 years had 1.5 to 3 times higher prevalence of poor SRH already 11–12 years prior to death compared to surviving controls. The risk estimates remained statistically significant even after adjusting for life-style related risk factors and diagnosed diseases. Prevalence of poor SRH before death was lowest among those aged ≥80 years and highest in 30–64 year-olds. In conclusion, men and women who subsequently die perceive their health worse already 11–12 years prior to death compared to their surviving controls.

## Introduction

The one-item assessment of self-rated health (SRH) is a global measure of health status that is widely used in surveys partly because of its simplicity and convenience. SRH has been shown to capture a wide range of health-related phenomena, such as chronic diseases, health behaviors, symptoms, functional limitations [1], and even immunological biomarker abnormalities [2], [3]. It is also considered an important tool to monitor population health and has been shown to be an independent predictor of subsequent health events, including functional decline [4], physician visits [5] and hospital episodes [6]. Research during the past three decades has also documented the association between poor SRH and increased risk of mortality [7], [8], [9] across different cultures [7], [10] and age groups [5], [11], .

The majority of prior investigations on the association between SRH and mortality have relied on SRH measurement at one time point, despite the fact that SRH likely changes over the course of long follow-up periods. In studies of mortality, the assessment of SRH at just one point in time is likely to introduce bias toward the null because of non-differential misclassification. To overcome this bias some studies have treated SRH as a time-varying variable [13], [14], and found that time-varying SRH is more strongly related to mortality compared to baseline-only measurement of SRH. However, even in these studies SRH has been measured at just two time points. Longitudinal studies with repeated measures focusing on within-individual changes in SRH remain sparse and very few studies have presented trajectories of SRH in different population subgroups [15], [16], [17], [18], [19], [20]. In these studies, however, the aim has been to characterize the different profiles of SRH over time rather than to examine it in respect to certain outcome of interest, such as mortality.

A better understanding of SRH trajectories at the end of life potentially may help to shed light on the mechanisms linking age, SRH, chronic conditions and death. To examine this issue, we used data from the Health and Retirement Study (HRS), a longitudinal cohort study of retirement and health among a representative sample of older people in the United States. The extraordinarily rich and complex data with repeated measurements provides an opportunity to examine the trajectories of SRH during the 12 years prior to death in different age groups of men and women utilizing a nested case-control design. Such data also allowed our examination of the role of common risk factors and diseases in explaining the differences in SRH between deceased cases and surviving controls.

## Methods

### Study design and participants

The Health and Retirement Study (HRS) is an ongoing cohort study of Americans, with interview data collected biennially on demographics, health behavior, health status, employment, income and wealth, and insurance status. The first cohort was interviewed in 1992 and every two years subsequently, with 5 additional cohorts added in the phases between 1994 and 2010. The full details of the study are described elsewhere [21], [22].

**Figure 1:**
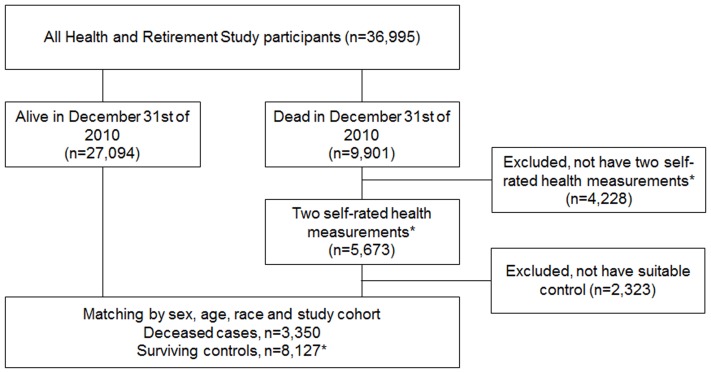
Flow chart of the study population. * At least two self-rated health measurements in the 12 years prior to death, one proximal (1–6 years prior to death) and one distal (7–12 years prior to death).

In this study we focused on the HRS participants who died between 1998 and 2010 (n = 9,901, Figure 1). We restricted inclusion in the study to those who had at least two SRH measurements in the 12 years prior to death, one observation proximal (1–6 years) and one distal (7–12 years) prior to death (n = 5,673). To compare SRH trajectories of those who died to surviving participants we utilized a nested case-control design with deaths being the cases (n = 3,350) and randomly selected survivors (at least one, but three at most) serving as controls for each case (n = 8,127). Controls were matched for sex, baseline age (5-year groups), race (White/Caucasian, Black/African American and Other) and study cohort. The trajectory of SRH for each individual was traced backwards for 12 years and they also had to have one proximal and one distal SRH measurement. The analytic sample consisted of 1,622 men and 1,728 women who died at a mean age of 73.9 (SD 8.5) years and 75.9 (SD 10.0) years, respectively. On average, deceased cases provided data on SRH at 5.1 (SD 1.0) timepoints while surviving controls provided data at 5.1 (SD 1.1) of the possible 6 waves.

### Ethical approval

Ethical approval for the HRS Study was obtained from the University of Michigan Institutional Review Board and the study has been conducted according to the principles expressed in the Declaration of Helsinki. Since the interviews were conducted by telephone, all respondents are read a confidentiality statement when first contacted, and they give verbal consent by agreeing to do the interview. This consent procedure was approved by the ethic committee.

### Measurement of self-rated health

At each study wave, the participants were asked to rate his or her health on a five-point scale ranging from 1 (excellent) to 5 (poor). For the purpose of this study SRH was dichotomized by categorizing response scores 1–3 (excellent, very good, good) as good health and scores 4–5 (fair and poor) as poor health. We used all available annual measurements in the 12-year timeframe before death.

### Mortality outcome

Mortality data on all participants was available in the HRS database. The HRS procedure to confirm the death and the timing of a participant's death relies on proxy informants, matching to the Social Security Death Index as well as matching to the commercial Insight databases to determine the vital status of all participants. These death records are periodically validated with the gold standard National Death Index (NDI). HRS routinely confirms death dates against the NDI report by comparison with the proxy reported death dates. For deceased participants, the month and year of death are recorded in the database. In the analyses we included deaths between 1 January 1998 and 31st December 2010.

### Covariates

There are two time-invariant variables: age at death and education. Age was categorized as 30–64 years, 65–79 years and ≥80 years. Education was categorized in three levels (low  =  less than high school, medium  =  high school or some college, and high  =  college and above). All other covariates were time-varying since they could change between study phases. Marital status was categorized as married/cohabiting, widowed and other possibilities. Non-housing financial wealth was divided into sex-specific tertiles.

Of the major risk factors, we had information on smoking, obesity and high blood pressure. Smoking status was defined as no, ex-smoker and current smoker. Body Mass Index (BMI) was calculated using reported height and weight, and categorized as normal weight BMI 18.5–24.9 kg/m^2^, overweight BMI 25–29.9 kg/m^2^, obese BMI≥30 kg/m^2^. High blood pressure was based on self-report of a doctor-diagnosed condition.

Chronic diseases were determined at each study wave by asking respondents: “Has a doctor ever told you that you have…?” Six chronic diseases were included: (1) heart disease (heart attack, coronary heart disease, angina, congestive heart failure, or other heart problems), (2) stroke, (3) chronic lung disease (chronic bronchitis or emphysema), (4) cancer (cancer or a malignant tumor of any kind except skin cancer), (5) diabetes (diabetes or high blood sugar) and (6) psychiatric condition.

### Statistical analysis

Characteristics of the study population are presented separately for men and women as well as by case and control status. Marital status and non-housing financial wealth are based on the last information prior to death for deceased cases and from the corresponding study phase for the surviving controls.

Trajectories of poor SRH in the 12 years preceding death were assessed using log-binomial regression analysis with generalized estimating equations (GEE) controlling for the intra-individual correlation between repeated measurements using an exchangeable correlation structure [23], [24], [25]. First, we plotted sex-specific SRH trajectories for all deceased cases and surviving controls. Second, we examined whether the trajectories were dependent on age groups and tested age group x time interaction terms for cases and controls. The interactions for deceased men (p<0.001) and women (p<0.001) were statistically significant and therefore the main results are shown by age groups.

To examine whether the prevalence of poor SRH among the deceased cases and surviving controls differed during the 12-year period, we compared their first (11–12 years before death) and last (1–2) SRH measurements. In addition, we also examined the change from first to last recorded SRH within cases and controls. The analysis was initially adjusted for education, wealth and marital status. The second set of models further adjusted for life-style related risk factors and the third set of models for chronic diseases. All covariates, except education, were used as time-varying using the closest available assessment corresponding to the self-reported health measurement. Results are presented as risk ratios (RR) and their 95% confidence intervals (CI).

Our sensitivity analysis addressed the possibility that including only those deceased participants for whom we could find an age, sex, race and cohort matched control may have affected the findings. We estimated the 12-year SRH trajectory by taking into account all those men and women who met the first inclusion criteria (one proximal and one distal SRH measurement (n = 5,089). The SAS 9.4 Statistical Package was used for all analyses (SAS Institute Inc., Cary, NC).

## Results

Characteristics before death of deceased cases and surviving controls (selected from the same study wave) are shown in Table 1. The average age at death was 73.9 years among male and 75.9 years among female cases and the respective age of the controls were 73.1 years and 75.4 years. Age, sex, race and cohort matched controls had higher level education and non-housing wealth compared to deceased cases (p for all <0.0001). In addition, deceased women were more often married or cohabiting than their controls (p = 0.01).

**Table 1:** Characteristics Before Death Among Deceased Cases and Surviving Controls*.

	Men			Women		
	Deceased cases	Surviving controls	p	Deceased cases	Surviving controls	p
N	1622	3827		1728	4300	
Age at death/last visit,%			0.04			0.45
30–64	13.0	13.8		12.9	13.6	
65–79	59.9	62.3		49.0	49.9	
≥80	27.1	23.9		38.2	36.5	
Race,%			0.38			0.85
White	80.6	82.1		77.7	78.3	
Black	15.5	13.5		19.0	18.6	
Other	3.9	3.8		3.3	3.1	
Education,%			<.0001			<.0001
Less than high school	39.1	30.2		41.3	30.8	
High school	43.0	47.5		48.9	55.4	
College and above	18.0	22.3		9.7	13.8	
Non-housing financial wealth,%			<.0001			<.0001
Lowest tertile	44.7	30.2		43.3	30.5	
Middle tertile	28.2	32.6		29.5	32.4	
Highest tertile	27.1	37.3		27.1	37.1	
Marital status,%			<.0001			<.0001
Married or cohabiting	71.9	79.9		38.1	47.2	
Widowed	14.2	9.9		46.0	39.0	
Other possibilities	13.9	10.2		15.9	13.9	

* Characteristics are selected from the same study wave for cases and controls.

**Figure 2:**
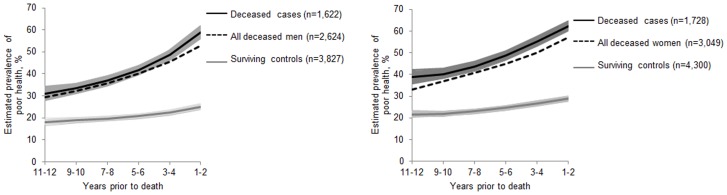
Estimated prevalence with 95% confidence intervals of poor self-rated health during the 12 years prior to death in deceased cases and surviving controls. Panel A: men, Panel B: women. Dotted line indicates the trajectory for all deceased men/women in the HRS study with one proximal and one distal self-rated health measurement including also those who did not have a matched control.

Figure 2 shows the trajectories of poor SRH in the 12 years preceding the death in deceased cases and surviving controls. In men and women, the prevalence of poor SRH across the 12-year period was higher among deceased cases compared to controls (p for all <0.0001). In deceased men, comparing their first SRH measurement (11–12) to their last SRH measurement (1–2), the prevalence of poor SRH doubled (RR 1.88, 95% CI 1.72–2.06). Among surviving controls the increment from first to last SRH measurement was 1.5-fold (RR 1.48, 95% CI 1.36–1.62). In women, the increment from first to last SRH measurement was slightly less than in men (cases RR 1.56, 95% CI 1.45–1.68 and controls RR 1.44, 95% CI 1.34–1.55). Compared to the surviving controls, the prevalence of poor SRH increased more steeply among male and female cases of all ages (interaction for age*group p<0.0001 for men and p = 0.01 for women).

For the sensitivity analysis Figure 2 also shows the trajectory of all deceased men and women who had one proximal and one distal SRH measurement including those who did not match to a control. The trajectory of all deceased men closely trailed the trajectory of deceased males fulfilling the case definition. The trajectory of all deceased women was slightly lower but parallel with the trajectory of deceased female cases. This was because many of the oldest participants did not anymore have surviving controls available. For those 2,323 cases who were excluded, due to not having a matched control, the average death age was 86.5 years among males and 89.5 among females.

**Figure 3:**
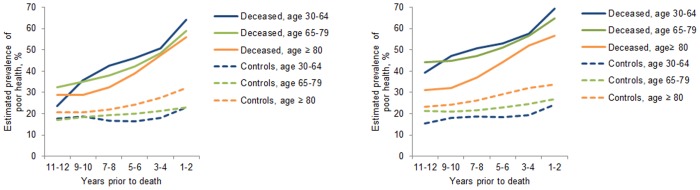
Estimated prevalence of poor self-rated health during the 12 years prior to death in deceased cases and surviving controls by age groups. Panel A: men, Panel B: women.

Figure 3 shows the trajectories of poor SRH in the 12 years preceding the death among deceased cases and surviving controls in the three different age groups. In men and women, the prevalence of poor SRH across the 12-year period among cases was lowest among those aged 80 years and older, whereas among controls it was lowest among those aged 30–64 years. The risk ratios for poor SRH comparing deceased cases and surviving controls at 11–12 and 1–2 years prior to death are shown in Table 2 (men) and Table 3 (women). In men aged 65–79 and 80 years and older, those who subsequently died reported between a 1.5 to 2.0-fold higher prevalence of poor SRH already 12 years prior to death compared to their surviving controls (RR 1.92, 95% CI 1.66–2.24 and RR 1.48, 95% CI 1.15–1.92) (Table 2). Among men aged 30–64 years, there was no statistically significant difference in prior poor SRH comparing cases and controls (RR 1.61, 95% 0.93–2.81), but the association was significant two years later i.e. 10 years prior to death (RR 1.85, 95% 1.31–2.61). Adjustment for sociodemographic factors and lifestyle-related risk factors slightly attenuated the risk estimates, which were further attenuated after accounting for chronic diseases. In the fully adjusted model, the difference between cases and controls remained significant for both older age groups.

**Table 2:** Risk Estimates (RR) and Their 95% Confidence Interval (CI) for Men's Poor Self-Rated Health Comparing Deceased Cases to Surviving Controls at 11–12 and 1–2 Years Prior to Death.

	Model 1			Model 2			Model 3			Model 4		
Age group	RR	95% CI		RR	95% CI		RR	95% CI		RR	95% CI	
30–64 years												
11–12 years prior	1.61	0.93	2.81	n/a*			n/a			n/a		
1–2 years prior	2.79	2.29	3.40	n/a			n/a			n/a		
65–79 years												
11–12 years prior	1.92	1.66	2.24	1.76	1.52	2.04	1.71	1.48	1.99	1.49	1.29	1.71
1–2 years prior	2.55	2.32	2.80	2.31	2.10	2.54	2.26	2.05	2.48	1.69	1.53	1.86
≥80 years												
11–12 years prior	1.48	1.15	1.92	1.42	1.10	1.82	1.40	1.09	1.80	1.28	1.01	1.64
1–2 years prior	1.65	1.45	1.88	1.56	1.37	1.78	1.55	1.36	1.77	1.28	1.12	1.47

*Notes*: Model 1 is adjusted for age; Model 2 is additionally adjusted for education, wealth and marital status; Model 3 is additionally adjusted for BMI, smoking and blood pressure; Model 4 is additionally adjusted for heart disease, stroke, lung disease, cancer, diabetes, and psychiatric disease.

* model fails to converge.

**Table 3:** Risk Estimates (RR) and Their 95% Confidence Interval (CI) for Women's Poor Self-Rated Health Comparing Deceased Cases to Surviving Controls at 11–12 and 1–2 Years Prior to Death.

	Model 1			Model 2			Model 3			Model 4		
Age group	RR	95% CI		RR	95% CI		RR	95% CI		RR	95% CI	
30–64 years												
11–12 years prior	3.33	2.17	5.10	2.72	1.78	4.16	2.54	1.67	3.86	2.11	1.41	3.16
1–2 years prior	2.84	2.37	3.41	2.32	1.93	2.78	2.12	1.77	2.54	1.54	1.28	1.85
65–79 years												
11–12 years prior	2.07	1.81	2.37	1.80	1.58	2.05	1.69	1.48	1.91	1.43	1.27	1.62
1–2 years prior	2.38	2.18	2.60	2.07	1.89	2.26	1.97	1.80	2.16	1.46	1.33	1.61
≥80 years												
11–12 years prior	1.50	1.26	1.79	1.47	1.23	1.75	1.44	1.21	1.72	1.36	1.15	1.61
1–2 years prior	1.59	1.43	1.76	1.54	1.39	1.70	1.53	1.38	1.69	1.30	1.17	1.44

*Notes*: Model 1 is unadjusted; Model 2 is adjusted for education, wealth and marital status; Model 3 is additionally adjusted for BMI, smoking and blood pressure; Model 4 is additionally adjusted for heart disease, stroke, lung disease, cancer, diabetes, and psychiatric disease.

In women aged 30–64 years those who died reported more than three times higher prevalence of poor SRH compared to surviving controls 12 years prior to death (RR 3.33, 95% CI 2.17–5.10) and the risk ratio remained relatively stable across the 10-year time period (Table 3). Among women aged 65–79 years and 80 years and older the risk ratios of mortality among those with poor SRH were between 1.5–2.0 fold higher, comparing cases and surviving controls. Adjustment for sociodemographic factors, lifestyle-related risk factors and chronic diseases attenuated but did not eliminate the observed differences between deceased cases and surviving controls in all age groups.

As expected, there was a marked difference between cases and controls in the prevalence of poor SRH in the wave immediately preceding death for both sexes (Table 2 and Table 3). However, the prevalence ratio of poor health was inversely related to age. Poor SRH was reported nearly three times more often by deceased men (RR 2.79, 95% CI 2.29–3.40) and women (RR 2.89, 95% CI 2.37–3.41) aged 30–64 compared to their surviving controls. The risk ratio was slightly lower in men and women aged 65–79 years and much lower among 80 years and older men (RR 1.65, 95% 1.45–1.88) and women (RR 1.59, 95% CI 1.43–1.76) compared to surviving controls. Adjustment for sociodemographic factors, lifestyle-related risk factors and chronic diseases attenuated but did not eliminate the observed differences between deceased cases.

## Discussion

Our analysis of the individual-level repeat data from the US Health and Retirement Study provides two insights into the SRH trajectories prior to death in middle-aged and older US adults. First, those who died reported 1.5 to 3 times higher prevalence of poor SRH even 11–12 years prior to their death compared to their age, sex and race matched surviving controls. Large differences between deceased cases and surviving controls were observed among women dying at age of 30–64 years. Second, trajectories of poor SRH among deceased cases differed across age groups and sex, being lowest in men and women aged 80 years and older and highest in women aged 30–64 years. In surviving controls, poor SRH increased linearly with age, the prevalence being highest among those aged 80 years and older.

To examine factors that might explain the differences between deceased cases and surviving controls 12 years prior to death, we separately adjusted for lifestyle factors and chronic diseases. In men and women dying in 30–64 and 65–79 years of age, obesity, smoking and high blood pressure partly explained the observed difference. However, lifestyle factors had a minor role in the oldest age group. Further adjustment for chronic diseases explained large part of the difference between deceased cases and surviving controls in all age groups, but all risk estimates remained statistically significant even in the fully adjusted models. These results confirm earlier findings of the nature of SRH by capturing some unobserved factors predisposing to death in addition to clinically observed diseases and risk factors [2], [7], [26].

To our knowledge this is the first study to present age-related SRH trajectories 12 years prior to death for deceased cases and their surviving controls. In surviving controls the prevalence of poor SRH increased over time in all age groups, slightly more in the oldest age group, which is in accordance with the previously reported age-related SRH trajectories [27]. Interestingly, the trajectories of poor SRH among deceased cases and surviving controls were furthest apart for those aged 30–64 years and closest to each other in those in the oldest age group. This unexpected finding is most likely due to a selective survival bias at older ages, i.e. people with very poor SRH have already died prior to reaching age of 80 years. It is also possible that the level of expectations regarding “good” health decreases with age, and at any given level of SRH more health problems are tolerated [28].

SHR is a simple but comprehensive measure of global health and several other factors besides disease risk factors and comorbid diseases may determine people's perceptions as suggested by recent meta-analysis [29]. First, it is possible that SRH captures a wide array of mortality-related physiological and pathological characteristics, such as increased inflammation [2], [3], which we could not examine in this study. Moreover, it has been suggested that positive self-ratings reflect a general optimistic disposition to life. Good perceived health may be particularly important at more advanced ages when physical and psychological impairments are common [30]. Finally, SRH also captures some other “private” or unobserved characteristics that determine health status, such as family history, health behavior, pre-illness symptoms, as well as social and psychological resources [31], which may partly explain the differences observed already in the middle-aged group.

The main strengths of the study include the prospective longitudinal design with biennial information on SRH during 12 years of follow-up. The results can be generalized to the US adult population due to the nationally representative sample and because our inclusion criteria did not seem to cause bias in the SRH trajectories. In addition, by using the nested case-control design we were able to examine the “true” differences between SRH trajectories in deceased cases and surviving controls during 12-year time window since the matching took care of the confounding role of age, sex and race, which all are found to influence of SRH measurement.

The limitations of the study also need to be acknowledged. Our analysis examined the associations of SRH trajectories with all-cause mortality, since cause-specific mortality data was not available. Further studies are warranted to examine whether SRH trajectories differ according to causes of death. Second, the HRS ascertained a limited list of self-reported comorbid conditions, thus no information was available for many chronic illnesses such as Parkinson's disease which can impact on perceived health. It is also possible that we failed to capture prodromal illnesses (i.e. diagnoses of diseases which had not yet been confirmed by a physician at the time of answering the surveys).

To conclude, data from a population-based sample of US adults showed that men and women who subsequently die perceive their health worse already 11–12 years prior to death compared to their surviving controls. In clinical practice single-item SRH can serve as a comprehensive, patient-centered and inexpensive “screening” tool to identify persons at increased risk of deteriorating health and premature death.
